# Porous Alumina Membrane-Based Electrochemical Biosensor for Protein Biomarker Detection in Chronic Wounds

**DOI:** 10.3389/fchem.2020.00155

**Published:** 2020-03-06

**Authors:** Gayathri Rajeev, Elizabeth Melville, Allison J. Cowin, Beatriz Prieto-Simon, Nicolas H. Voelcker

**Affiliations:** ^1^Future Industries Institute, University of South Australia, Mawson Lakes, SA, Australia; ^2^Monash Institute of Pharmaceutical Sciences, Monash University, Parkville, VIC, Australia; ^3^Department of Electronic Engineering, Universitat Rovira i Virgili, Tarragona, Spain; ^4^Commonwealth Scientific and Industrial Research Organisation (CSIRO) Manufacturing, Clayton, VIC, Australia; ^5^Melbourne Centre for Nanofabrication, Clayton, VIC, Australia; ^6^Materials Science and Engineering, Monash University, Clayton, VIC, Australia

**Keywords:** porous alumina, electrochemical biosensor, flightless detection, chronic wound, immunosensing

## Abstract

A label-free electrochemical detection platform for the sensitive and rapid detection of Flightless I (Flii) protein, a biomarker of wound chronicity, has been developed using nanoporous anodic alumina (NAA) membranes modified with Flii antibody recognition sites. The electrochemical detection is based on the nanochannel blockage experienced upon Flii capture by immobilized antibodies within the nanochannels. This capture impedes the diffusion of redox species [[Fe(CN)_6_]^4−/3−^] toward a gold electrode attached at the backside of the modified NAA membrane. Partial blockage causes a decrease in the oxidation current of the redox species at the electrode surface which is used as an analytical signal by the reported biosensor. The resulting biosensing system allows detection of Flii at the levels found in wounds. Two types of assays were tested, sandwich and direct, showing <3 and 2 h analysis time, respectively, a significant reduction in time from the nearly 48 h required for the conventional Western blot assay. Slightly higher sensitivity values were observed for the sandwich-based strategy. With faster analysis, lack of matrix effects, robustness, ease of use and cost-effectiveness, the developed sensing platform has the potential to be translated into a point-of-care (POC) device for chronic wound management and as a simple alternative characterization tool in Flii research.

## Introduction

Flightless I (Flii) is a member of the gelsolin family of actin-remodeling proteins which plays a significant role in wound healing and as an intermediate protein linking the cytoskeleton to various signaling pathways (Kopecki and Cowin, 2008). Flii is characterized by a combination of two functional protein domains which are the leucine-rich repeat (LRR) domain and the gelsolin-like domain, consisting of 1,256 amino acids in total with a weight of 143,672 Da. The Cowin group has investigated the role of Flii in the wound healing process and found that the over-expression of Flii impairs wound healing, while its attenuation improves it by increasing cellular proliferation, epithelial migration and enhanced wound contraction (Cowin et al., 2007; Adams et al., 2008, 2009). To this end, Flii can be a potential therapeutic target for improving wound healing and also an important wound biomarker for monitoring the wound progress. Flii neutralizing monoclonal antibody (FnAb) developed against the LRR domain of Flii has been shown to improve wound healing in both *in vitro* and *in vivo* models (Jackson et al., 2012; Kopecki et al., 2012; Ruzehaji et al., 2012, 2014). Hence there is a growing research interest in understanding the functions of Flii in chronic wound management. Currently, the common techniques used for Flii detection are Western blotting (Cowin, 2006; Cowin et al., 2007; Jackson et al., 2012) and immunohistochemistry (Cowin, 2006; Lin et al., 2011; Jackson et al., 2012). These techniques are non-quantitative, time consuming, tedious and involve multiple steps. To this end, development of a simpler and faster biosensor able to detect Flii would be of great interest. Such sensor could become the basis of a POC device for chronic wound care. Several sensors to assess wound healing progression have been developed, targeting infection, pH, oxygen, uric acid, hemoglobin, and broad-spectrum proteases (Dargaville et al., 2013; Edwards et al., 2013; Sharp, 2013; Krismastuti et al., 2014, 2015a, 2017; Ochoa et al., 2014; Salvo et al., 2015; Jankowska et al., 2017; RoyChoudhury et al., 2018). These sensors have not reached yet the stage of deployment in the clinic due to the challenges involved, such as the complex wound environment with hundreds of proteins present in a wide pH range.

Porous materials offer outstanding features for biosensor development. Among these, nanoporous anodic alumina (NAA) is a popular material used for biosensing applications due to its highly ordered structure, tuneable pore geometry, unique optical and electrical properties, thermal and mechanical stability, biocompatibility, chemical resistance, and large surface-to-volume ratio (Md Jani et al., 2013). Several electrochemical and optical biosensing platforms have been developed using NAA (Santos et al., 2013; Krismastuti et al., 2015b; Rajeev et al., 2018). NAA-based detection platforms offer certain advantages over other nanoporous material-based detection platforms (such as those based on porous silicon and porous silica). Since NAA is fabricated by self-ordering electrochemical anodization, it results in uniform, and parallel nanochannels. The uniformity and homogeneity of NAA structures contribute to enhance their reproducibility, leading to sensing platforms able to provide accurate and reliable sensing results. NAA also has high chemical, thermal and mechanical stability, and excellent biocompatibility, overcoming some of the limitations encountered by other types of porous materials (Rajeev et al., 2018; Reta et al., 2018). An additional advantage of NAA worth to harness when developing biosensors relates to the chemical properties of NAA, particularly the large amount of hydroxyl groups present on its porous surface, which allow facile biofunctionalization.

Electrochemical detection is a powerful transduction method due to its ability to detect target molecules with high sensitivity and short detection time. Most importantly, electrochemical transduction facilitates miniaturization into smart POC devices. To this end, we combine these attributes of electrochemical sensing with the versatile properties of NAA to develop a sensing platform for Flii detection. The NAA membrane-modified electrode functionalized with antibodies within the nanochannels effectively increases the number of available biorecognition elements compared to flat electrodes and thus offers improved sensing performance.

The partial or complete blockage of NAA nanochannels due to analyte specific binding to capture probes immobilized within the pores has been used as a sensing strategy for direct and label-free electrochemical detection of various biomolecules (Vlassiouk et al., 2005; Koh et al., 2007; Nguyen et al., 2009, 2012; de la Escosura-Muñiz and Merkoçi, 2010, 2011; de la Escosura-Muñiz et al., 2013; Espinoza-Castañeda et al., 2015; Tang et al., 2016; Reta et al., 2018). A blocking event impedes the diffusion of an electroactive species added in solution to the electrode surface which is measured as a reduction in oxidation current. Modifying electrodes with an NAA membrane facilitates implementing this versatile sensing strategy.

NAA membranes with highly ordered cylindrical pores can be fabricated by electrochemical anodization of high purity Al (Masuda and Fukuda, 1995). This allows to tune the geometrical features of the membrane such as nanochannel diameter (10–400 nm) and membrane thickness (from few 100 nm to hundreds of μm). These characteristics can be effectively used to tune the sensitivity of biosensors based on nanochannel blockage by designing nanochannels with suitable geometrical features considering the size of the biomolecules involved.

Herein, we present a novel electrochemical biosensor for Flii detection using NAA membranes. Since isolated or recombinant whole Flii protein is not readily available, initially we used a Keyhole Limpet Hemocyanin (KLH)-conjugated peptide containing the active sequence of Flii protein for developing a model immunosensor for Flii detection. Partial nanochannel blockage due to specific binding of analyte within the pores is used as sensing strategy. Flii secreted by wounded keratinocytes was successfully detected. We compared the sensing performance of the developed biosensor using commercially available and home-made geometry-tuned NAA membranes. Our research proves that designing membranes with pore geometry to suit the analyte of interest significantly improves biosensor sensitivity. This simple and fast sensing platform is promising as a diagnostic tool in chronic wound care.

## Experimental

### Materials

High purity aluminum (Al) foil (99.998%) with 0.5 mm thickness was purchased from Jomar Life Research (Australia). Commercial NAA membrane filters (Whatman Anodisc filters, 13 mm in diameter, 0.1 μm pore diameter, 60 μm thickness) were purchased from Interpath services (Australia). Oxalic acid (98%), 3-(triethoxysilyl) propyl isocyanate (95%), phosphate buffered saline (PBS) tablets, potassium ferrocyanide [K_4_[Fe(CN)_6_]], potassium ferricyanide [K_3_[Fe(CN)_6_]], and Dulbecco's Modified Eagle's Medium (DMEM) were purchased from Sigma-Aldrich (Australia). Hydrogen peroxide (30%) was purchased from Chemsupply (Australia). Mouse monoclonal anti-Flii antibodies (FnAb) raised against the N-terminus of the LRR domain of the human Flii protein was developed in house (Jackson et al., 2012). Anti-human IgG antibodies used for control experiments were purchased from Sapphire Bioscience (Australia). Mouse monoclonal anti-Flii antibodies were obtained from Santa Cruz Biotechnology (USA).

Custom made KLH-conjugated Flii peptide was purchased from Mimotopes (Australia). Gold-coated microscope slides were purchased from Telic company (USA).

### NAA Membrane Fabrication and Morphological Characterization

NAA membranes were fabricated by 2-step electrochemical anodization. Al sheet was cut into 16 mm discs, degreased by sonicating in acetone for 10 min followed by washing in Milli-Q water and dried under nitrogen gas stream prior to anodization. A chemical polishing of the Al discs was carried out to remove the surface roughness by boiling in a 15:85 mixture of 68% HNO_3_ and 85% H_3_PO_4_, respectively, at 70°C and neutralized by immersing in 1 M NaOH for 20 min as reported elsewhere (Alam et al., 2011). This technique is used as an alternative pre-treatment to electropolishing which is the most commonly used technique, to avoid the use of perchloric acid that requires dedicated fume hoods and highly efficient heat removal systems. Chemically polished Al samples were rinsed with copious amounts of Milli-Q water. Self-ordered porous alumina was fabricated by a two-step anodization procedure reported elsewhere (Masuda and Fukuda, 1995; Masuda, 2005). Briefly, anodization was performed in a custom-built anodization cell with an in-built cooling system connected to a recirculating cooler (Julabo F250) and a DC system power supply (Agilent N5751A). The first anodization step was done by immersing the polished Al discs in 0.3 M oxalic acid and applying a voltage of 40 V at 5°C for 20 h to obtain a long-range self-ordering of the pores. This step creates a disordered porous layer at the top, which is removed by treating in a mixture of 6 wt.% H_3_PO_4_ and 1.8 wt.% H_2_CrO_4_ at 70°C. This is followed by the second anodization which is done under the same conditions as the first anodization but for 24 h. In order to obtain the NAA membrane, remaining Al at the backside was removed by metal displacement chemistry by treating in a solution of CuCl_2_/HCl. The removal of barrier layer at the backside was done by chemical etching in 5% H_3_PO_4_ for 90 and 120 min at room temperature to obtain different pore diameters.

Morphology of the samples was characterized using scanning electron microscopy (SEM), and SEM image analysis was performed using ImageJ.

### NAA Surface Modification and FTIR Characterization

In order to immobilize the bioreceptors (FnAb) on the pore walls, a standard silanization chemistry was done using 3-(triethoxysilyl) propyl isocyanate. NAA membrane was treated in 30% H_2_O_2_ solution at 70°C for 1 h to obtain fresh hydroxyl groups and to remove any organic contaminants. After this, the hydroxylated membrane was dried under a mild nitrogen flow and baked in oven at 60°C for 2 h to remove any remaining water content since the silanes are highly reactive to any residual water. Then, the membrane with active hydroxyl groups was immersed in a 5% solution of isocyanate silane in dry toluene in a sealed reaction vessel under nitrogen atmosphere. This reaction was carried out for 2 h under constant shaking at room temperature and inert atmosphere, which resulted in isocyanate groups (-N=C=O) on the membrane surface. After reaction, the sample was removed and washed with fresh dry toluene and dried under a nitrogen stream. Finally, the functionalized membrane was incubated with 50 μg ml^−1^ antibody solution in 0.1 M PBS (pH 7.4) for 2 h under constant shaking. After incubation, the membrane was washed thoroughly with copious amounts of PBS, and stored in PBS at 4°C until used for sensing experiments.

Fourier transform infrared (FTIR) spectroscopy was used to characterize the surface after each step of surface modification. FTIR spectra were obtained using a Vertex 70 Hyperion microscope Bruker in reflectance mode (Bruker Optics, Germany). Background spectra, taken on a gold-coated glass slide, and sample spectra were recorded over the range of 650–1,000 cm^−1^ at a resolution of 4 cm^−1^, an aperture size of 3 mm and averaging 64 scans. Data were analyzed using OPUS 7.2 spectroscopy software (Bruker Optics, Germany).

### Electrochemical Biosensing of Flii Peptide Conjugate

The antibody-modified NAA membrane mounted on a gold-coated glass slide (Au/NAA-Ab) acts as the biosensing platform. Electrochemical experiments were conducted using a three-electrode system in a Teflon cell in which Au/NAA-Ab acted as the working electrode, a silver/silver chloride (CH instruments, USA) was used as the reference electrode and a platinum wire (CH instruments, USA) was used as the counter electrode. The electrochemical analyser used for the experiments was obtained from CH Instruments (model 600D series, USA).

The immunosensor was incubated with 500 μL of Flii peptide solution in 0.1 M PBS (pH 7.4) over a concentration range of 0.5–50 μg ml^−1^ for 1 h. After washing with PBS, 700 μL of a redox species solution containing 2 mM of K_4_[Fe(CN)_6_] and 2 mM K_3_[Fe(CN)_6_] in 0.1 M PBS were added to perform electrochemical measurements. Differential pulse voltammetry (DPV) was used as detection technique in which a series of regular voltage pulses were applied over a range of potential from −0.3 to 0.8 V and the oxidation current of the redox species was measured at 0.19 V. DPV measurements were done before and after incubating each Flii peptide solution. Each experiment was performed in triplicate. Control experiments were done using an NAA membrane modified with non-specific antibody to assess the non-specific binding of the analyte. Similar experiments were performed in Dulbecco's Modified Eagle's Medium (DMEM) spiked with Flii peptide for matrix effects studies.

### Flii Secretion in Wounded HaCat Media

Human keratinocytes (HaCat) were used in *in vitro* assays. Keratinocytes were cultured to confluency, scratch wounded using a modified cell scraper and further incubated in DMEM for 10 h. Conditioned medium was collected and concentrated using Vivaspin 20 centrifugal concentrators. Similarly, medium exposed to unwounded keratinocytes was collected and concentrated for use in control experiments.

### Western Blotting

Western blotting was performed on conditioned medium to validate the presence of Flii. A standard Western blotting procedure was used. Briefly, samples were run on 12.5% SDS-PAGE gels at 100 V for 1 h and then transferred into nitrocellulose at 100 V for 1 h (Bio-Rad Laboratories, USA). The membranes were blocked with 12% skimmed milk for 15 min with shaking at RT, and then incubated with anti-Flii antibody solution containing 5% skimmed milk mix overnight at 4°C. The membrane was washed two times in Tris-Tween 20-buffered saline (TTBS) and incubated with a horseradish peroxidase (HRP)-labeled anti-mouse secondary antibody for 1 h at RT. After this, the membrane was washed twice in TTBS and once in Tris-buffered saline (TBS). HRP detection was performed on this stained membrane by Super Signal West Femto Maximum Sensitivity Substrate (Pierce Biotechnology, USA) and capture using GeneSnap analysis program (SynGene, USA).

### Electrochemical Biosensing of Flii Protein Secreted From Wounded Cells

Conditioned medium collected from scratch wounded keratinocytes containing secreted Flii, as confirmed by Western blot results, was analyzed using the developed immunosensor and the protocol previously described (section Electrochemical Biosensing of Flii Peptide Conjugate). After incubating the conditioned medium containing Flii on the immunosensor surface, a sandwich assay was performed by further incubation of a detection mouse monoclonal anti-Flii antibody (Santa Cruz Biotechnology, USA) solution. This additional step was performed to compensate for the smaller size of the Flii protein compared to the peptide conjugate previously used for the optimization of the immunosensor's analytical performance. Control medium from unwounded cells was analyzed following the same protocol.

## Results and Discussion

### Characterization of the NAA Membrane-Modified Electrode as Electrochemical Biosensing Platform

**Scheme 1:**
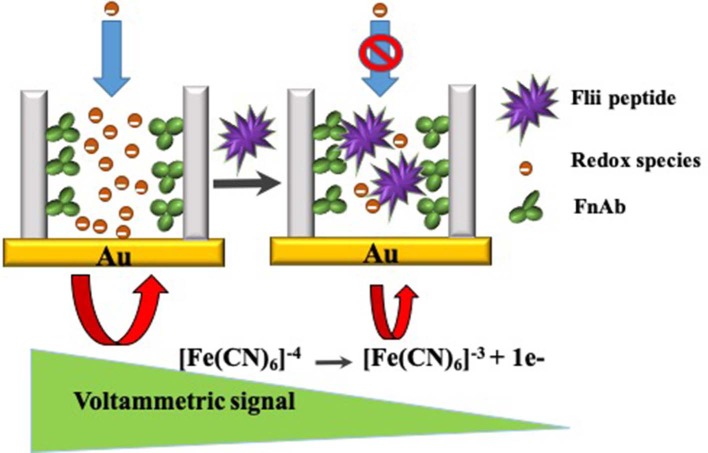
Schematic representation of NAA electrochemical sensing based on pore blockage.

In this study, label-free detection of Flii using NAA membranes is attributed to the partial steric blockage of the membrane nanochannels upon binding to the immobilized antibodies. The sensing principle for the detection is given in Scheme 1. NAA biosensors based on the same sensing mechanism have previously been explored using commercially available Whatman Anodisc membrane filters. These commercial membranes are available in 3 pore diameters (20, 100, and 200 nm) and a single membrane thickness (60 μm). While this is a convenient choice, the ability to tune the sensing performance of the device is limited since the extent of blockage due to steric hindrance is related to the size of the analyte and diameter of the modified nanochannels.

In our work, a model sensing platform for Flii detection was initially developed using a KLH-conjugated peptide which contains the active sequence of Flii protein (H-CKLEHLSVSHN-OH). The peptide conjugate has an overall molecular weight of 390 kDa and an average diameter of 30 nm (Kopecki and Cowin, 2008). In our sensing platform, FnAb is used as the bioreceptor which can selectively capture the conjugated peptide as well as Flii protein secreted from chronic wounds.

**Figure 1:**
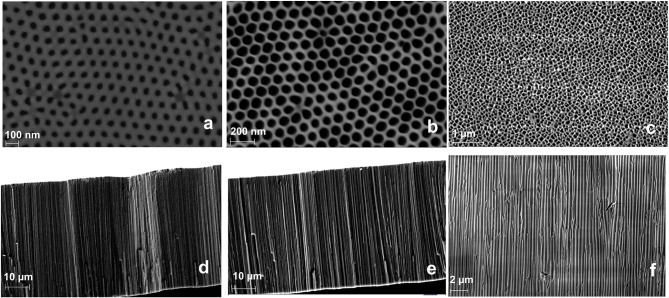
SEM micrographs of top-view NAA membranes fabricated at 40 V using 0.3 M oxalic acid and pore opened for 90 min **(a)**, 120 min **(b)**, and commercial Anodisc membrane **(c)**; and the respective cross-section images showing vertical pore channels **(d–f)** (in same order).

Here, we compare the analytical performance of a Flii immunosensor prepared with either commercially available NAA membrane filters or home-built NAA membranes with tuned pore characteristics. For this purpose, apart from the 100 nm commercial Anodiscs, we fabricated NAA membranes with smaller pore diameters (keeping the membrane thickness the same for all) to prove the ability to fine tune the sensitivity by fabricating membranes of optimum pore diameter. This was achieved by using different barrier removal times during the membrane fabrication. Pore diameter was calculated by image analysis on ImageJ. Nanochannel diameters of 45 ± 5 and 62 ± 5 nm were achieved at barrier removal times of 90 and 120 min, respectively. Top-view and cross-section SEM images of fabricated and commercial membranes are given in Figure 1. The nanochannel diameter increases as the pore widening time increases, as seen in the images. SEM images show that the fabricated samples have self-ordered, uniform and high-density pores throughout the surface, while the Anodiscs showed non-uniform heterogeneously distributed pores with an average pore diameter of 102 ± 4 nm. Fabricated membranes showed a more homogeneous distribution of pores in contrast to the Anodiscs.

### Characterization of NAA Membrane Surface Modification

**Figure 2:**
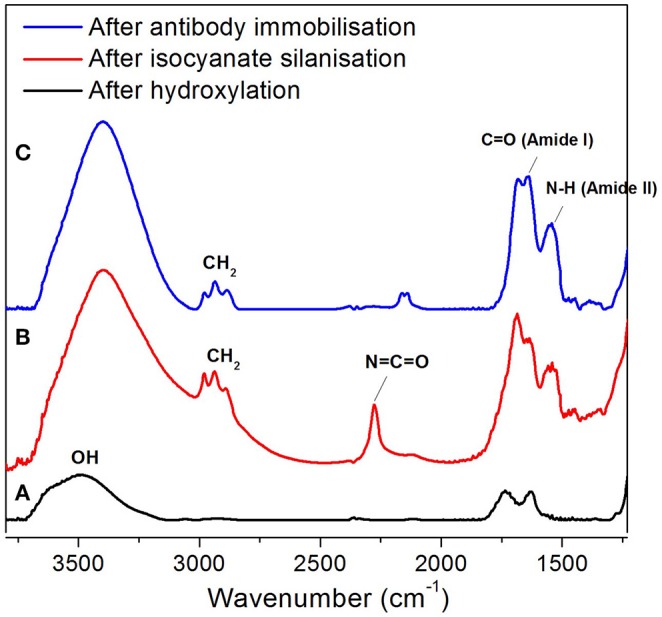
FTIR spectra of NAA membrane at various surface modification steps, after hydroxylation **(A)**, silanization **(B)**, and antibody immobilization **(C)**.

FTIR (reflectance mode) was used to confirm the surface modification steps of the NAA membranes. After hydroxylation, the FTIR spectrum showed a broad peak at 3,500 cm^−1^ which corresponds to the presence of hydroxyl groups on the NAA surface (Figure 2A). In the spectrum shown in Figure 2B, a characteristic band that was found at 2,250 cm^−1^ after reacting the hydroxylated membrane with 3-isocyanatopropyl-triethoxy silane representing N=C=O stretching vibration mode confirms the successful silanization and presence of isocyanate groups on the surface. Bands at 2,925 cm^−1^ and 2,854 cm^−1^ were assigned to the stretching vibration modes of the aliphatic CH_2_ groups in the silane. In this same spectrum, bands at 1,550 cm^−1^and 1,650 cm^−1^ were present which are characteristic of N-H bending and C=O stretching vibration modes, attributed to the fast hydrolysis undergone by some of the isocyanate groups upon reaction with residual water in the solvent or water vapor in the atmosphere. Figure 2C confirms successful immobilization of antibodies via the disappearance of the isocyanate band at 2,250 cm^−1^ and the intense bands at 1,550 cm^−1^ (N-H bending vibration mode) and 1,650 cm^−1^ (C=O stretching vibration mode) from the peptide bonds formed between the antibodies and the isocyanate groups.

### Label-Free Electrochemical Detection of Flii Peptide Conjugate

As described earlier, partial nanochannel blockage as a result of the interaction between the analytes and the bioreceptors immobilized on the NAA surface is exploited here as detection strategy. The blockage impedes the diffusion of redox species toward the gold electrode. The binding of Flii to the immobilized antibodies is measured as a reduction in the oxidation current of the redox species added in solution. Initial experiments were done using conjugated Flii peptide to study the effect of pore diameter tuning on the sensor's sensitivity. In this study, the sensor response from commercial (100 nm) and home-built (45 and 65 nm) membranes was compared. Dose response curves of different immunosensors were plotted as normalized current vs. log concentration of analyte. Normalized current, used to allow comparison of different sensing platforms, was calculated using the following formula:

(1) $${\text{I}}_{\text{nor}}=\frac{\left({\text{I}}_{0}-{\text{I}}_{\text{p}}\right)}{{\text{I}}_{0}}*100$$

where, I_nor_ is the normalized current change, and I_0_ and I_p_ are the current intensity values before adding the analyte and after analyte incubation for 1 h, respectively.

**Figure 3:**
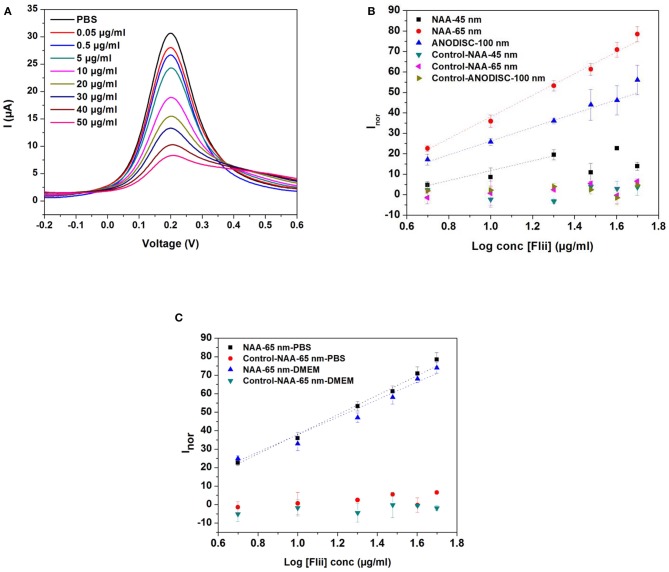
**(A)** DPVs for increasing concentrations of KLH-conjugated Flii peptide in PBS for immunosensors prepared with FnAb-modified membranes of 65 nm pore diameter; **(B)** dose response curves for KLH-conjugated Flii peptide detection using Flii and control biosensors fabricated with membranes of average pore diameter of 45, 65, and 100 nm; **(C)** dose response curves obtained by using Flii and control biosensors fabricated with 65 nm-pore diameter membranes for KLH-conjugated Flii peptide detection in PBS and DMEM medium. Error bars were calculated from three independent experiments. Control sensors were prepared under the same conditions as the Flii biosensors but using non-specific antibodies.

DPV curves for detection of the Flii conjugate using sensors prepared with 65 nm pore diameter membranes over a concentration range of 0.5–50 μg ml^−1^ and dose response curves for Flii conjugate detection using various sensing platforms and control surfaces are shown in Figures 3A,B. It can be seen from the DPV curves that the current intensity decreased with the increasing concentration of the KLH-conjugated peptide. Sensing performance was compared by determining the sensitivity of the immunosensors calculated as the slope of the linear curve fit and is summarized in Table 1. All experiments were performed three times to check reproducibility. Results show that the 65 nm-pore diameter membranes provide biosensors with the best sensing performance with a sensitivity of 52.9 ml μg^−1^ compared to the 100 nm-pore diameter commercial membranes and the 45 nm-pore diameter fabricated membranes which have sensitivities of 33.7 ml μg^−1^ and 24.2 ml μg^−1^, respectively. We attribute this trend to the 65 nm pores having the most favorable and highest extent of steric hindrance during binding events. We chose 45 and 65 nm-pore diameter membranes from the consideration of the size of Flii-KLH conjugate and the bioreceptors. To provide the highest sensitivity, the extent of blockage upon analyte binding should be maximized by choosing pores that are small enough to be almost completely blocked, but also large enough to facilitate the diffusion of the protein during incubation. Our results showed that 45 nm pores are too small and do not efficiently allow the binding of Flii conjugate to the bioreceptors within the nanochannels. However, we found from our experiments that the 100 nm pores are not as efficient in pore blockage as the 65 nm pores, because they are too large to provide the maximum extent of pore blockage. This result confirms the value of using fabricated membranes with suitable nanochannel diameter for developing sensing platforms rather than relying on commercially available membranes for attaining the best sensing performance. Control sensing surfaces prepared by immobilizing non-specific antibodies showed no response to the presence of Flii conjugate (Figure 3B) which confirmed the specificity of the FnAb and the absence of non-specific adsorption of the Flii conjugate.

**Table 1:** Comparison of the sensing performance of different immunosensors for detection of Flii conjugate in PBS (calculated based on the response curve in Figure 3B).

**Pore diameter (nm)**	**Sensitivity (ml μg^**−1**^)**	**LOD (μg ml^**−1**^)**	**Linear range (μg ml^**−1**^)**	**Linear equation**	***R*^**2**^**
45	24.19	1.64	5–20	*y* = 24.19x−12.45	0.9544
65	52.89	1.01	5–50	*y* = 52.89x−14.88	0.9937
100	33.69	1.04	5–50	*y* = 33.69x−7.49	0.9806

Once demonstrated the performance of the model immunosensor for Flii detection using Flii conjugate, Flii protein secreted in wounded HaCat cells grown in DMEM was used for *in vitro* assays in the next set of experiments. So before performing these experiments in DMEM, it was important to demonstrate that there are no matrix effects from DMEM itself. For this purpose, sensing in DMEM spiked with conjugated Flii peptide was performed using the 65 nm-pore diameter membrane-modified immunosensor (Figure 3C). Slope deviation between the response toward KLH-conjugated Flii peptide in buffer and in medium was <5% which indicated that pronounced matrix effects were absent.

### Electrochemical Detection of Flii Protein Secreted in *in vitro* Wound Model and Signal Amplification via Sandwich Assay

**Figure 4:**
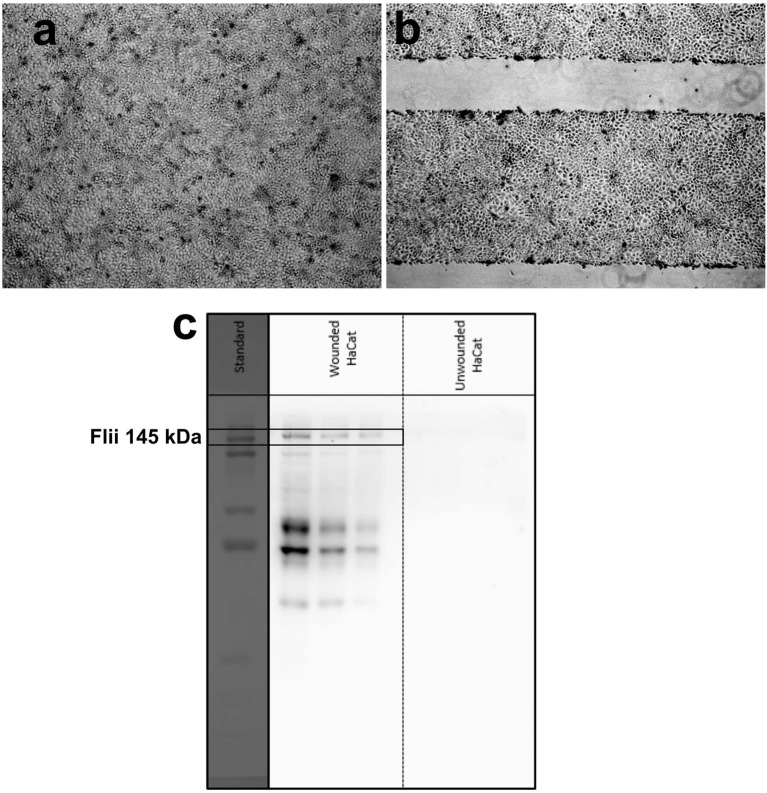
Brightfield microscopy images of **(a)** unwounded and **(b)** wounded HaCat cells at confluency (lines without cells in **(b)** correspond to deliberately induced scratches in the confluent HaCat cells); **(c)** Western blot for three dilutions (from left: 1.0X, 0.6X, and 0.2X) of media collected from wounded and unwounded keratinocytes.

Detection of Flii protein in chronic wounds is of great significance in wound analysis. A rapid Flii biosensor is not only promising as a clinical tool in wound management but also as a fast and simple characterization tool in Flii research. To validate the usability of the developed sensing platform for Flii analysis in wound models, Flii secreted from wounded keratinocytes was used. First, a Western blot was performed for three dilutions (1.0X, 0.6X, and 0.2X) of conditioned medium to confirm the presence of Flii and to compare with the sensing response of the developed immunosensor. Three dilutions of conditioned media from wounded and unwounded cells were tested to check first with the unwounded cells that no significant matrix effects were observed, and if so, to show that even diluting the samples to minimize the interfering effects still allows Flii detection. Brightfield microscopy images of HaCat cells before and after wounding are given in Figures 4a,b. Lines without cells in Figure 4b correspond to deliberately induced scratches in the confluent HaCat cells. Bands at 145 kDa in Figure 4c correspond to Flii protein in wounded cells.

DPV measurements were carried out before and after 1 h incubation of conditioned medium using the 65 nm-pore diameter membrane-modified immunosensors. Control experiments were performed using medium collected from unwounded cells. Since the size of Flii is smaller than that of the KLH-conjugated Flii peptide used in the previous experiments, medium incubation and thus Flii binding was followed by a signal amplification step using a sandwich assay based on a second 1 h incubation with detection Flii antibody. All measurements were done in triplicate to verify the reproducibility.

**Figure 5:**
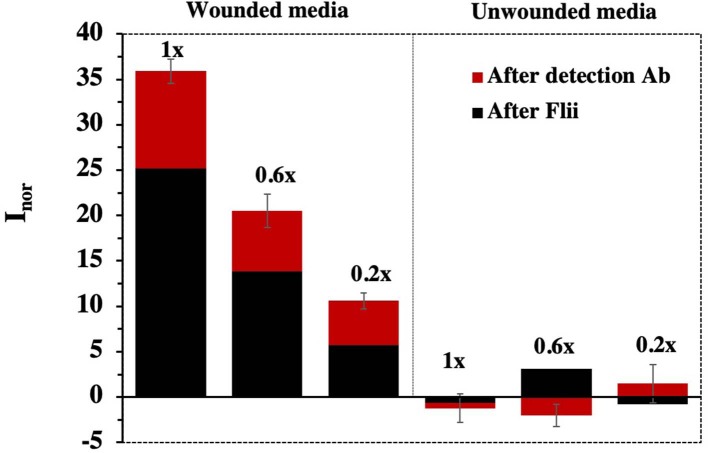
Sensing response for concentrated wounded HaCat media containing Flii protein obtained using biosensors fabricated with NAA membranes of 65 nm pore diameter. Error bars were calculated from three separate experiments.

As it can be observed in Figure 5, direct detection of Flii was possible even for the highest dilution of the conditioned medium containing Flii tested (1:5). Figure 5 also shows the signal amplification achieved using the immunosensor based on a sandwich assay. Control experiments performed using media collected from unwounded cells did not show any significant signal which confirms that the current changes observed were a result of specific binding between Flii protein present in the media collected from wounded cells and the antibodies immobilized in the nanochannels. This indicates that our sensor has the capacity to detect Flii in wound models without any significant matrix effects from interfering species that could be present in the conditioned media.

Sensitivity was determined as the slope of the response curve. As expected, between the two Flii biosensors, slightly higher sensitivity was observed for the sandwich-based platform (32.4 ml μg^−1^ and 24.3 ml μg^−1^, respectively). This Flii biosensor seems to be a fit-for-purpose analytical tool for Flii detection in wound models and greatly reduces the analysis time from almost 2 days for a Western blot to <3 h.

## Conclusions

We have successfully developed for the first time a simple, direct and rapid electrochemical biosensor for highly sensitive detection of Flii protein in chronic wounds. Flii protein is a key biomarker of chronic wounds. However, the potential to develop a wound diagnostic device based on Flii detection has not been explored yet. Techniques used currently involve tedious conventional methods, such as Western blotting, which are time-consuming, expensive due to the reagents used, and have a very low sensitivity. Moreover, these techniques only determine the presence or absence of Flii protein in wounds (Jackson et al., 2012; Kopecki et al., 2012; Ruzehaji et al., 2014). Our efforts in the reported work are to fill this gap and develop a new sensing technology for Flii protein detection in chronic wounds. Therefore, we present a nanochannel based electrochemical biosensor for Flii protein detection based on pore-blockage on NAA membranes which were carefully designed by considering parameters such as pore diameter. Sensing is based on nanochannel blockage on antibody modified home-built alumina membranes upon Flii binding which is translated as a reduction in the oxidation current of the redox species added to the measuring solution.

Two home-made membranes with a pore diameter of 45 and 65 nm, and one commercial membrane with a pore diameter of 100 nm were used to compare the sensing performance. Our studies prove that the performance of the developed biosensor can be improved significantly by tuning the pore geometry to suit the size of target analyte. Geometry tuned, home-fabricated membranes with pore diameter of 65 nm showed enhanced sensing performance with a sensitivity of 52.9 ml μg-1 compared to the 100 nm-pore diameter commercially available alumina membranes and the 45 nm-pore diameter fabricated membranes which have sensitivities of 33.7 and 24.2 ml μg-1, respectively. The developed biosensing system was further used for the detection of Flii secreted in *in vitro* wound models. Direct and label-free detection of secreted Flii was demonstrated. However, to compensate for the small size of the Flii protein compared to the KLH Flii-peptide used for the biosensor optimization, a sandwich immunoassay approach was applied to amplify the measured signal. Although the sandwich-based strategy included an additional 1 h-incubation step, the analysis time was still <3 h, greatly shortening the almost 48 h required for conventional methods like Western blotting.

Our biosensor is promising not only as a biomarker detection platform for chronic wound management, but also as an easy-to-use characterization tool in Flii research. The high stability/robustness and versatility of home-made membranes combined with high sensitivity and short detection time make this platform a powerful tool in clinical as well as research fronts.

## Data Availability Statement

All datasets generated for this study are included in the article/supplementary material.

## Author Contributions

The experiments presented in this work were designed by AC, NV, and BP-S. GR and EM conducted the experimental work. GR, EM, AC, NV, and BP-S discussed the results obtained from the experiments. GR wrote the manuscript and the last version was revised by all authors. All authors read and approved the final manuscript.

### Conflict of Interest

The authors declare that the research was conducted in the absence of any commercial or financial relationships that could be construed as a potential conflict of interest.
